# Deepwater Horizon oil spill exposures and nonfatal myocardial infarction in the GuLF STUDY

**DOI:** 10.1186/s12940-018-0408-8

**Published:** 2018-08-25

**Authors:** Jean Strelitz, Lawrence S. Engel, Richard K. Kwok, Aubrey K. Miller, Aaron Blair, Dale P. Sandler

**Affiliations:** 1 Department of Epidemiology, Gillings School of Global Public Health, University of North Carolina at Chapel Hill, Chapel Hill, NC USA; 2 National Institute of Environmental Health Sciences, NIH, DHHS, Research Triangle Park, NC USA; 3 National Cancer Institute, NIH, DHHS, Bethesda, MD USA

**Keywords:** Oil spill, Deepwater Horizon, Myocardial infarction, Disaster epidemiology

## Abstract

**Background:**

Workers involved in the response and clean-up of the 2010 *Deepwater Horizon* oil spill faced possible exposures to crude oil, burning oil, dispersants and other pollutants in addition to physical and emotional stress. These exposures may have increased risk of myocardial infarction (MI) among oil spill workers.

**Methods:**

Gulf Long-term Follow-up (GuLF) STUDY participants comprise individuals who either participated in the *Deepwater Horizon* response efforts or registered for safety training but were not hired. Oil spill-related exposures were assessed during enrollment interviews conducted in 2011–2013. We estimated risk ratios (RR) and 95% confidence intervals for the associations of clean-up work characteristics with self-reported nonfatal MI up to three years post-spill.

**Results:**

Among 31,109 participants without history of MI prior to the spill, 77% worked on the oil spill. There were 192 self-reported MI during the study period; 151 among workers. Among the full cohort, working on the oil spill clean-up (vs not working on the clean-up) and living in proximity to the oil spill (vs further away) were suggestively associated with a possible increased risk of nonfatal MI [RR: 1.22 (0.86, 1.73) and 1.15 (0.82, 1.60), respectively]. Among oil spill workers, working for > 180 days was associated with MI [RR for > 180 days (vs 1–30 days): 2.05 (1.05, 4.01)], as was stopping working due to heat [RR: 1.99 (1.43, 2.78)]. There were suggestive associations of maximum total hydrocarbon exposure ≥3.00 ppm (vs < 0.30 ppm) [RR: 1.69 (0.90, 3.19)] and working on decontaminating oiled equipment (vs administrative support) [1.72 (0.96, 3.09)] with nonfatal MI.

**Conclusion:**

This is the first study to assess the associations between oil spill exposures and MI. Results suggest that working on the spill for > 180 days and stopping work due to heat increased risk of nonfatal MI. Future research should evaluate whether the observed associations are related to specific chemical exposures or other stressors associated with the spill.

## Background

The 2010 *Deepwater Horizon* disaster led to the largest marine oil spill in history. The spill began April 20, 2010, following an explosion on the *Deepwater Horizon* drilling rig in the Gulf of Mexico which killed 11 oil rig workers and resulted in the release of over 200 million gallons of crude oil [1]. Individuals involved in the oil spill response and clean-up faced potential exposures to crude oil, burning oil, dispersants and cleaning agents, in addition to physical stress related to oil spill work tasks [2, 3]. Exertion, heat exposure, psychosocial stress, and chemical exposures associated with the oil spill may have impacted the cardiovascular health of clean-up workers, in addition to the socioeconomic impacts of the oil spill on residents of communities affected by the spill [4–6].

Research on past oil spills has shown some acute health consequences among clean-up workers and individuals from affected communities, including anxiety and post-traumatic stress [7], eye and skin irritation [8], and lower respiratory tract symptoms [9] up to one year following oil spill exposure. In several studies, longer durations of oil spill work were associated with acute respiratory symptoms, including among US Coast Guard personnel deployed to work on the *Deepwater Horizon* spill [10], and *Hebei Spirit* oil spill workers [11]. Persistent respiratory symptoms were observed among clean-up workers 5 years following the *Prestige* oil spill [12]. It is unclear what specific oil spill-related exposures or physical factors drive these associations, but longer work durations may be indicative of a larger chemical exposure burden, as well as increased physical or psychosocial stress related to the oil spill. Despite the reported associations between oil spill work and persistent respiratory symptoms, no research has assessed incidence of other chronic health outcomes, such as cardiovascular events, among oil spill-exposed populations [13].

Many workers involved in the oil spill response and clean-up faced physical stress from high ambient temperatures and the manual labor that was required for many clean-up tasks, which could contribute to coronary events. Workplace stressors such as noise, along with co-exposures to volatile organic chemicals, may increase risk of hypertension [14], a primary risk factor for coronary heart disease (CHD) [15]. Vigorous physical exertion increases risk of acute myocardial infarction (MI), particularly among adults who do not habitually participate in physical activity and who have atherosclerotic disease [16]. Tasks such as carrying or lifting equipment, working outdoors in high heat, and other labor associated with oil spill work may have created an environment with increased risk of triggering acute cardiovascular events or exacerbating existing coronary disease conditions among workers [4, 17].

Aside from the physical stress of oil spill work, workers faced possible exposures to volatile organic compounds (VOCs) and other chemicals in fresh and weathered oil, combustion products from burning oil and flaring of natural gas, emissions from the equipment and machinery used during the clean-up, and chemical dispersants [3, 18, 19]. Thus, depending on the nature, location, and timing of work being performed, exposures may include a complex mixture of particulate matter, polycyclic aromatic hydrocarbons, and VOCs [2]. Particulate matter is known to impact cardiovascular health, and even short-term increases in ambient particulate concentrations increase risk of cardiovascular events and overall mortality [20, 21]. Particulate levels during the oil spill clean-up were elevated in coastal communities and around clean-up sites, compared to typical ambient levels in these regions [22], and pollutant concentrations varied spatially over the course of the spill [18]. Volatile organic compounds, such as benzene, released from fresh oil may have also impacted cardiovascular health. Chronic occupational exposure to benzene has been linked to increases in hypertension and is also associated with hematologic changes [23]. While exposures to benzene and other VOCs potentially increase risk of adverse health effects, the impact of such exposures on cardiovascular events in relation to oil spills has not been explored.

An oil spill workers’ tasks and work locations during the clean-up may have been important indicators of chemical exposures related to the oil spill. Workers on boats, barges, and oil rigs were more often exposed to the volatile constituents present in fresh crude oil, while workers located on land or who performed clean-up along shorelines were more likely to be exposed to weathered oil with less volatile content [19]. Furthermore, workers who were tasked with burning oil deposits or who worked near burning oil may have had greater exposures to particulate matter.

Most oil spill workers were residents of the Gulf Coast region and may have experienced the socioeconomic impacts of the oil spill in addition to clean-up exposures. Local industries including fishing and tourism were disrupted for months in the wake of the oil spill [24]. Surveys of Gulf Coast residents showed decreases in income and increases in job loss following the spill [25], which may have contributed to psychosocial stress in these communities. Adverse mental health symptoms were also elevated among individuals living in or adjacent to a county where oil appeared during the spill [26]. Though community-level exposures may have included relatively little oil exposure or other chemical exposures related to the spill, the economic impacts on the region may have resulted in psychosocial stress. Psychosocial stress can impact risk of cardiovascular disease by accelerating formation and progression of atherosclerotic plaques [27]. Acute stress elevates blood pressure and may impact cardiac arrhythmia and myocardial ischemia, which can result in onset of an acute MI or contribute to an increased risk of a future CHD event by driving atherosclerotic progression and worsening cardiovascular disease states [4].

We assessed whether living in areas socioeconomically impacted by the oil spill, or involvement in oil spill work and the resulting clean-up related exposures, may be associated with risk of myocardial infarction (MI) in the 1 to 3-year period following the spill. Using data from the Gulf Long-Term Follow-up Study (GuLF STUDY) [3], we examined the relationship of clean-up work involvement and living in areas that were likely affected socioeconomically by the spill with risk of self-reported nonfatal MI within 1–3 years after the *Deepwater Horizon* oil spill.

## Methods

### Data collection

The GuLF STUDY is a prospective cohort study of individuals who worked for at least one day on, or who trained for but did not work on, the response and clean-up of the 2010 *Deepwater Horizon* oil spill. Participants in the study include individuals who completed mandatory oil spill safety training in order to take part in the oil spill response and clean-up as well as government workers and oil professionals. Details of the study design and participant enrollment have been described previously [3].

Recruitment for the GuLF STUDY began in March 2011, 11 months after the start of the oil spill, and continued through May 2013. Eligible participants were at least 21 years old and lived in the United States (US) at the time of enrollment. From a list of 62,803 presumably unique names with sufficient contact information, a total of 32,608 participants were enrolled into the study. Of these, we excluded 999 individuals who completed a Vietnamese language abbreviated version of the questionnaire which did not collect complete information on oil spill clean-up jobs, leaving 31,609 participants for this analysis.

During the enrollment telephone interview, conducted in English or Spanish, participants were asked to provide demographic and lifestyle information, details about tasks they performed during the oil spill response and clean-up, and information on their personal health history, including any diagnoses of MI. If the participant reported ever having an MI, they were asked the month and year of their first diagnosis. If they could not recall the month and year, they were asked their age at diagnosis. We excluded 495 study participants (366 clean-up workers, 129 non-workers) who reported an MI occurring prior to the oil spill, and 5 participants who reported an MI with unknown timing, resulting in a final sample size of 31,109.

Information on oil spill clean-up activities was assessed retrospectively during the enrollment telephone interview, 1–3 years after the spill. Participants were asked their home residence location, work locations during the oil spill response and clean-up, dates of oil spill work, and detailed information on their tasks, and contact with oil, dispersants and other chemicals during the oil spill. The study protocol was approved by the Institutional Review Board (IRB) of the National Institute of Environmental Health Sciences and this analysis was approved by the IRB at the University of North Carolina at Chapel Hill.

### Defining outcomes, exposures, and covariates

The outcome of interest was any incident self-reported diagnosis of a first nonfatal MI. MI considered in this analysis were those occurring after an individual began clean-up work, or, for non-workers, from the start of the oil spill on April 20, 2010 until the enrollment interview. Therefore, the risk period for an incident MI was the three-year period following the start of the oil spill.

In this analysis we evaluated the complex and varied exposures that workers faced during the oil spill. This included participation in clean-up work and duration of clean-up work, clean-up tasks, heat stress, and exposures to crude oil and burning oil during the clean-up work. To consider the impact of community level stress resulting from the spill, we assessed associations with living in, or adjacent to, a county or parish with coastline oiled during the spill. We considered both coastal and adjacent counties because these areas were most likely to have been impacted socioeconomically by the oil spill. Living in, or adjacent to, a county oiled during the spill was also a predictor of post-spill adverse mental health outcomes [26].

The exposures of interest in this analysis were defined as participation in clean-up work (yes, no); duration of oil spill clean-up work (categorized based on the distribution of work duration as 1–30 days, 31–90 days, 91–180 days, > 180 days); highest exposure clean-up job (response, operations support work, clean-up on the water, clean-up on land, decontamination, administrative support – classified hierarchically (highest to lowest) by likely level of exposure to total hydrocarbons as an indicator of exposure to oil spill chemicals [19]); maximum overall total hydrocarbon (THC) exposure during oil spill work (< 0.30 ppm, 0.30–0.99 ppm, 1.00–2.99 ppm, ≥3.00 ppm) as estimated from a job exposure matrix [3, 19] as described below; potential work exposure to burning crude oil (yes, no), which was derived from work task and location information; ever having had to stop clean-up work activities due to heat (yes, no); and residential proximity to the oil spill (“direct or indirect proximity”: resident of a county or parish that had coastline oiled from the spill or was adjacent to a county with oiled coastline; “away from the spill”: elsewhere in the Gulf states or broader US). All exposure data were self-reported or were derived from self-reported information, aside from maximum THC exposure estimates which also incorporated information on hydrocarbon concentrations from personal exposure monitors.

A job exposure matrix was used to assign workers to maximum total hydrocarbon (THC) exposure categories based on their highest exposure task during the clean-up. The job exposure matrix (JEM) was created using data from personal exposure monitor measurements of VOCs, including THC, collected during the oil spill response and clean-up. Exposure ranges were determined and ordinal estimates for level of THC exposure were assigned to exposure groups defined by vessel or vessel type (for clean-up work on the water), job type and task, and time period of clean-up work and ultimately linked to data from participant questionnaires to estimate individual exposure levels. Levels of exposure to THC served as a proxy for oil spill chemical exposure and was assigned to each self-reported work task using the JEM. Most participants reported multiple work tasks during clean-up, with some reported during the same time period. For this analysis, workers were characterized by their maximum overall THC exposure across all self-reported jobs/tasks [19].

Information on other covariates including age, gender, ethnicity, cigarette smoking, height and weight, income, and education were ascertained via self-report during the enrollment telephone interview.

### Estimating risk of heart attack

We used log binomial regression to estimate risk ratios (RR) for the association of each exposure with nonfatal MI, with separate models for each association of interest. Analyses focused on worker/non-worker status and residential proximity to the oil spill included the full cohort, while analyses of specific clean-up work characteristics (work duration, job type, THC exposure, burning oil exposure, heat exposure) were among workers only, using workers with the lowest exposure or no exposure as the referent group. Each regression model adjusted for age at enrollment (20–29, 30–39, 40–49, 50–59, 60–64, ≥65 years), gender (male, female), cigarette smoking (current, former, never), body mass index (BMI) (< 25, 25–29.9, ≥30 kg/m^2^), maximum education attainment (less than high school, high school diploma/GED, some college/2-year degree, 4+ year college graduate) and residential proximity to the oil spill (direct/indirect, away from the spill). We were unable to control for finer categories of smoking than current/former/never because there was a substantial amount of missing data for pack-years of smoking among former smokers. When modeling associations with residential proximity to the oil spill, stopping work due to heat, highest exposure job, and working near burning oil, we controlled for clean-up work duration in addition to the other confounders.

We evaluated the impact of heat stress on MI in several ways. We compared those who reported ever having to stop clean-up work due to heat to those who did not report this. We also carried out sensitivity analyses in which we adjusted for this measure of potential heat stress in the other exposure models to determine if adjusting for heat stress changed any associations with nonfatal MI. To differentiate potentially more acute heat stress-related MI events from any longer-term impacts of exposures on MI risk, we compared associations with nonfatal MI in two time periods - the active clean-up period up beginning April 20, 2010 until the end of 2010, and 2011 through the end of follow-up. Most clean-up activities had ceased by the end of 2010 and subsequent MI events were unlikely to be acutely due to clean-up-related heat stress.

### Effect measure modification

We assessed the presence of effect measure modification by smoking or residential proximity to the oil spill, using stratified analyses and product terms. Since smoking is a strong risk factor for MI, associations between oil spill exposures and MI could be more apparent among nonsmokers or there could be synergistic effects between smoking and oil spill chemicals. We considered residential proximity to the oil spill as a potential effect measure modifier to evaluate the possibility of a synergistic effect among clean-up workers living in areas affected by the spill, who may have experienced socioeconomic stress related to the oil spill in addition to chemical/physical oil spill-related exposures. Smoking status was defined as current/former smoker vs. never smoker, and residential proximity to the spill remained coded as direct/indirect proximity to the spill vs. away from the spill. Estimates for the exposure/outcome relationships were generated using log binomial regression models after stratifying by the modifier. To assess effect measure modification on the multiplicative scale, a product term between the modifier and main exposure was included in each model. We tested the statistical significance of modification of the risk ratio on the multiplicative scale using the likelihood ratio test (LRT) to compare models including and not including the product term, with alpha = 0.05.

## Results

Among the 31,109 study participants without a previous MI, there were 192 first MI cases occurring after the start of the oil spill; 151 were among oil spill workers and occurred after they began oil spill-related work. As seen in Table 1, 81% of participants were male, 57% were ≥ 40 years old, 66% were white, and 24% reported having graduated from college. More than half of all study participants reported living in a county or parish proximal to the oil spill and 77% of participants worked on the oil spill response and clean-up for at least one day (workers). Among those who worked on the spill, 88% worked for over 30 days. Maximum overall estimates of THC exposure ranged from 0 ppm to 23.24 ppm (data not shown), with 14% of workers having maximum THC exposure ≥3.00 ppm during clean-up (Table 2).

**Table 1:** Nonfatal MI by participant demographic characteristics. GuLF STUDY, 2010–2013 (*N* = 31,109)

	Nonfatal MI (*N* = 192) (%)	Total N (*N* = 31,109) (%)
Worked on oil spill clean-up		
Yes	151 (78.6)	23,933 (77.2)
No (non-workers)	41 (21.4)	7070 (22.8)
Missing		106 (0.3)
Gender		
Male	182 (94.8)	24,949 (80.5)
Female	10 (5.2)	6052 (19.5)
Missing		108 (0.3)
Age category (years)		
20–29	4 (2.1)	6214 (20.1)
30–39	20 (10.4)	7299 (23.6)
40–49	56 (29.2)	7593 (24.6)
50–59	64 (14.8)	6807 (22.0)
60–64	19 (15.1)	1746 (5.7)
≥ 65	29 (15.1)	1243 (4.0)
Missing		207 (0.7)
Ethnicity		
White	116 (60.4)	20,246 (65.7)
Black	49 (25.5)	7349 (23.8)
Asian	2 (1.0)	320 (1.0)
Other/multi-racial	25 (13.0)	2921 (9.5)
Missing		273 (0.9)
Hispanic		
Yes	12 (6.3)	2078 (6.7)
No	180 (93.8)	28,845 (93.3)
Missing		186 (0.6)
Education completed		
Less than high school	57 (29.7)	4950 (16.0)
High school diploma/GED	64 (33.3)	9257 (30.0)
Some college/2 year degree	54 (28.1)	9228 (29.9)
4+ year college graduate	17 (8.9)	7472 (24.2)
Missing		202 (0.6)
Income		
≤ $20,000	75 (43.6)	8102 (29.1)
$20,001 To $50,000	53 (30.8)	8890 (32.0)
More Than $50,000	44 (25.6)	10,831 (38.9)
Missing		3286 (10.6)
Residential location		
Direct/indirect proximity	140 (72.9)	18,914 (61.0)
Other residence	52 (27.1)	12,089 (39.0)
Missing		106 (0.3)
Cigarette smoking		
Current	62 (32.6)	9237 (30.0)
Former	69 (36.3)	6574 (21.4)
Never	59 (31.1)	14,979 (48.6)
Missing		319 (1.0)

**Table 2:** Nonfatal MI and oil spill clean-up characteristics among workers. GuLF STUDY, 2010–2013 (*n* = 24,006)

	Nonfatal MI (*N* = 151) (%)	Total N (*N* = 24,006) (%)
Work duration		
1–30 days	11 (7.3)	2936 (12.3)
31–90	46 (30.5)	7518 (31.4)
91–180	56 (37.1)	8216 (34.3)
> 180	38 (25.2)	5263 (22.0)
Missing		73 (0.3)
Maximum exposure job		
Response Work	20 (13.2)	4387 (18.3)
Operations Work	39 (25.8)	4289 (17.9)
Clean-up on Water	21 (13.9)	3699 (15.5)
Decon Work	33 (21.9)	3509 (14.7)
Clean-up on Land	18 (11.9)	3572 (14.9)
Administrative Support Work	20 (13.2)	4477 (18.7)
Missing		73 (0.3)
Potentially exposed to burning/flaring oil and gas		
Yes	13 (9.0)	2106 (8.9)
No	132 (91.0)	21,328 (91.1)
Missing or unknown		572 (2.4)
Ever had to stop work due to heat		
No	72 (50.7)	14,142 (65.8)
Yes	70 (49.3)	7358 (34.2)
Missing		2506 (10.4)
Max overall THC exposure		
≥ 3.00 ppm	24 (15.9)	3387 (14.2)
1.00–2.99 ppm	49 (32.5)	7324 (30.6)
0.30–0.99 ppm	60 (39.7)	7836 (32.8)
< 0.30 ppm	18 (11.9)	5360 (22.4)
Missing		99 (0.4)

Adjusted risk ratios (RR) from the regression models for each of the oil spill exposures and nonfatal MI are shown in Table 3. Among the full cohort, there were suggestive, but not statistically significant, associations for working on the oil spill response and clean-up (vs. not working on clean-up) and nonfatal MI [RR = 1.22, 95% Confidence Interval = (0.86, 1.73)], and for living in direct/indirect proximity to the oil spill (vs. away from the spill) with risk of MI [RR = 1.15 (0.82, 1.60)] after adjusting for confounders.

**Table 3:** Associations between oil spill exposures and first nonfatal MI. GuLF STUDY, 2010–2013

	MI cases/n^a^	RR^b^ (95% CI)
	Workers and non-workers (*N* = 31,109)	
Worked on clean-up		
Yes	149/23399	1.22 (0.86, 1.73)
No	40/6872	ref
Residential proximity to oil spill^c^		
Direct/indirect	138/18487	1.15 (0.82, 1.60)
Away from the spill	51/11784	ref
	Clean-up workers only (*n* = 24,006)	
Maximum exposure job^d^		
Response Work	20/4344	0.71 (0.37, 1.35)
Operations Work	39/4250	1.24 (0.70, 2.21)
Clean-up on Water	20/3650	0.73 (0.38, 1.39)
Decontamination Work	33/3481	1.72 (0.96, 3.09)
Clean-up on Land	18/3532	0.93 (0.49, 1.78)
Administrative Support	19/4392	ref
Work duration		
> 180 days	38/5162	2.05 (1.05, 4.01)
91–180 days	56/8056	1.77 (0.93, 3.37)
31–90 days	44/7327	1.63 (0.84, 3.14)
1–30 days	11/2854	ref
Ever had to stop work due to heat^d^		
Yes	70/7248	1.99 (1.43, 2.78)
No	72/13912	ref
Worked near burning oil^d^		
Yes	13/2072	1.00 (0.56, 1.77)
No	136/21327	ref
Maximum overall THC exposure		
≥ 3.00 ppm	24/3323	1.69 (0.90, 3.19)
1.00–2.99 ppm	49/7183	1.26 (0.71, 2.21)
0.30–0.99 ppm	59/7675	1.81 (1.05, 3.14)
< 0.30 ppm	17/5192	ref

Residential proximity to the spill is defined as living in or adjacent to a county or parish with coastline that was oiled during the spill

^a^Total number of observations included in the adjusted model

^b^Risk ratios (RR) adjusting for age, gender, BMI, education, residential proximity to the oil spill, and smoking

^c^Models adjust for duration of clean-up work in addition to the confounders age, gender, BMI, education, and smoking

^d^Models adjust for duration of clean-up work in addition to the confounders age, gender, BMI, education, smoking and residential proximity to the oil spill

Among oil spill workers, work duration > 180 days (vs. 1–30 days) was associated with increased risk of nonfatal MI [RR = 2.05 (1.05, 4.01)], although the suggestive exposure-response trend across category of work duration was not statistically significant (*p*-value for linear trend test = 0.20). There was a positive association between ever having to stop clean-up work activities due to heat and risk of MI [RR = 1.99 (1.43, 2.78)]. There were no apparent associations with MI by job type, aside from a possible association with decontamination work [RR = 1.72 (0.96, 3.09)]. There was no association with working near burning oil [RR = 1.00 (0.56, 1.77)]. Maximum overall THC exposure ≥3.00 ppm (vs < 0.30 ppm) had a possible association with nonfatal MI [RR = 1.69 (0.90, 3.19)], but with no evidence of an exposure-response trend.

Adjusting for stopping work due to heat did not substantively change any of the associations of interest (results not shown). In an analysis restricted to the 135 nonfatal MI (114 among workers) that occurred from January 1, 2011 through the end of the study period (Table 4), the association with working on oil spill clean-up [RR = 1.83 (1.13, 2.94)] was stronger than what was observed among first MI diagnoses across the entire study period. Other associations were generally similar to those observed for the entire study period; however the results for the later time period lacked precision due to the small number of cases in some strata (Table 4). There were too few cases to evaluate MI risk specifically during the active clean-up period or to further stratify the risk period.

**Table 4:** Associations of oil spill exposure characteristics and nonfatal MI occurring after December 31st 2010. GuLF STUDY 2010–2013

	MI cases/ total N^a^	RR^b^ (95% CI)
	Among workers and non-workers (*N* = 30,946; 135 cases)	
Worked on clean-up		
Yes	112/23362	1.83 (1.13, 2.94)
No	20/6852	ref
Residential proximity to oil spill^c^		
Direct/indirect	95/18444	1.17 (0.79, 1.74)
Away from the spill	37/11770	ref
	Among clean-up workers only (*n* = 23,896; 114 cases)	
Maximum exposure job^d^		
Response Work	14/4305	0.61 (0.29, 1.27)
Operations Work	25/4200	0.97 (0.50, 1.89)
Clean-up on Water	17/3603	0.76 (0.38, 1.55)
Decontamination	26/3436	1.66 (0.87, 3.16)
Clean-up on Land	14/3484	0.88 (0.43, 1.82)
Administrative Support	16/4334	ref
Work duration		
> 180 days	33/5157	1.99 (0.98, 4.04)
91–180 days	38/8038	1.35 (0.67, 2.70)
31–90 days	31/7314	1.27 (0.63, 2.59)
1–30 days	10/2853	ref
Ever had to stop work due to heat^d^		
Yes	51/7201	1.85 (1.25, 2.73)
No	55/13827	ref
Worked near burning oil^d^		
Yes	9/2068	0.93 (0.47, 1.84)
No	103/21294	ref
Maximum overall THC exposure		
≥ 3.00 ppm	16/3315	1.34 (0.65, 2.76)
1.00–2.99 ppm	35/7169	1.05 (0.56, 1.96)
0.30–0.99 ppm	46/7662	1.65 (0.91, 2.99)
< 0.30 ppm	15/5190	ref

^a^Number of observations included in the fully adjusted model, by exposure status

^b^Risk ratios (RR) adjusting for age, gender, BMI, education, smoking and residential proximity to the oil spill

^c^Models adjust for duration of clean-up work in addition to the confounders age, gender, BMI, education, and smoking

^d^Models adjust for duration of clean-up work in addition to the confounders age, gender, BMI, education, smoking and residential proximity to the oil spill

Overall, associations with clean-up work and residential proximity were more evident among never-smokers than among ever-smokers though the stratified estimates mostly had overlapping confidence intervals. Working on the oil spill appeared to be associated with MI only among never-smokers (Table 5). Never-smokers had somewhat stronger associations of work duration with MI compared to the associations among smokers, although there was no evidence of an exposure-response trend. The LRT statistic showed that the product term improved fit for models of job type and maximum THC exposure.

**Table 5:** Modification of the associations between oil spill exposures and nonfatal MI among workers and non-workers by smoking status. GuLF STUDY, 2010–2013

	MI cases/n^a^	RR^b^ (95% CI)	MI cases/n^a^	RR^b^ (95% CI)	LRT^e^ *P*-value^*^
	Among workers and non-workers without prevalent MI (*N* = 31,109)				
	Ever smoker (*n* = 15,861)		Never smoker (*n* = 15,016)		
Worked on clean-up					
Yes	100/12043	0.99 (0.67, 1.47)	52/11359	1.98 (0.98, 4.03)	0.11
No	33/3533	ref	9/3341	ref	
Residential proximity to oil spill^c^					
Direct/indirect	97/10377	0.99 (0.67, 1.48)	45/8114	1.52 (0.84, 2.76)	0.26
Away from the spill	36/5199	ref	16/6586	ref	
	Among clean-up workers and responders without prevalent MI (*n* = 24,009)				
	Ever smoker (*n* = 12,510)		Never smoker (*n* = 11,701)		
Maximum exposure job^d^					
Response Work	14/2338	0.66 (0.30, 1.45)	7/1974	0.80 (0.28, 2.25)	< 0.01
Operations Work	30/2471	1.21 (0.60, 2.44)	10/1744	1.11 (0.42, 2.93)	
Clean-up on Water	11./1955	0.53 (0.23, 1.22)	9/1651	1.02 (0.38, 2.75)	
Decontamination	26/1856	1.74 (0.86, 3.51)	7/1587	1.20 (0.43, 3.39)	
Clean-up on Land	7/1764	0.47 (0.18, 1.20)	11/1724	1.89 (0.75, 4.72)	
Administrative Support	12/1659	ref	8/2679	ref	
Work duration					
> 180 days	24/2699	1.53 (0.71, 3.30)	14/2463	2.90 (0.83, 10.09)	0.21
91–180 days	37/4306	1.37 (0.66, 2.83)	20/3751	2.54 (0.76, 8.55)	
31–90 days	30/3647	1.34 (0.64, 2.80)	15/3681	2.16 (0.63, 7.43)	
1–30 days	9/1391	ref	3/1464	ref	
Ever had to stop work due to heat^d^					
Yes	53/4125	2.39 (1.58, 3.60)	18/3096	1.23 (0.68, 2.24)	0.07
No	42/7048	ref	30/6796	ref	
Worked near burning oil^d^					
Yes	10/1104	1.11 (0.57, 2.14)	4/969	0.98 (0.35, 2.74)	0.31
No	90/10939	ref	48/10390	ref	
Maximum overall THC exposure					
≥ 3.00 ppm	19/1822	1.67 (0.79, 3.55)	6/1502	1.43 (0.47, 4.34)	0.05
1.00–2.99 ppm	34/4073	1.13 (0.56, 2.26)	16/3111	1.32 (0.53, 3.32)	
0.30–0.99 ppm	36/4061	1.43 (0.73, 2.83)	23/3614	2.29 (0.97, 5.39)	
< 0.30 ppm	11/2071	ref	7/3122	ref	

^a^Number of observations included in the fully adjusted model, by exposure status

^b^Risk ratios (RR) adjusting for age, gender, BMI, education, and residential proximity to the oil spill

^c^Models adjust for duration of clean-up work in addition to the confounders age, gender, BMI, and education

^d^Models adjust for duration of clean-up work in addition to the confounders age, gender, BMI, education, and residential proximity to the oil spill

^e^*LRT* Likelihood Ratio Test. The LRT was used to assess statistical significance of an interaction term between the modifier variable and the main exposure variable

*Alpha = 0.05

There did not appear to be much heterogeneity of the observed associations after stratifying by residential proximity to the oil spill, although some risk estimates were qualitatively stronger among those living in proximity to the spill (Table 6). For example, the association for stopping work due to heat and MI was significant among those living in proximity to the spill but was somewhat weaker in magnitude, and less precise, among those living further away [2.10 (1.42, 3.10) and 1.53 (0.80, 2.95), respectively]. Those living in proximity to the spill had a somewhat stronger association for maximum overall THC exposure ≥3.00 ppm with MI compared to workers living further away from the spill [1.99 (0.88, 4.51) and 1.27 (0.47, 3.38), respectively]. The LRT statistics showed that including a product term for home proximity to the spill did not improve fit for any of these models (*p*-values all > 0.05), indicating no modification of the risk ratio on the multiplicative scale.

**Table 6:** Modification of the associations between oil spill exposures and nonfatal MI by residential proximity to the oil spill. GuLF STUDY, 2010–2013

	MI cases/ total n^a^	RR^b^ (95% CI)	MI cases/ total n^a^	RR^b^ (95% CI)	LRT^e^ *P*-value^*^
	Among workers and non-workers (*N* = 31,109)				
	Direct/indirect proximity (*n* = 18,984)		Away from the spill (*n* = 12,125)		
Worked on clean-up					
Yes	106/13801	1.16 (0.79, 1.70)	43/9598	1.32 (0.62, 2.81)	0.81
No	32/4686	ref	8/2186	ref	
	Among clean-up workers and responders only (*n* = 24,375)				
	Direct/indirect proximity (*n* = 14,140)		Away from the spill (*n* = 9866)		
Maximum exposure job^c^					
Response Work	15/2439	0.83 (0.37, 1.87)	5/1872	0.58 (0.20, 1.66)	0.31
Operations Work	29/2836	1.35 (0.65, 2.80)	10/1378	1.06 (0.42, 2.68)	
Clean-up on Water	17/2400	0.82 (0.40, 1.82)	3/1206	0.46 (0.12, 1.68)	
Decontamination	25/2366	1.88 (0.89, 3.95)	8/1077	1.33 (0.50, 3.49)	
Clean-up on Land	11/2128	0.87 (0.37, 2.06)	7/1360	1.02 (0.38, 2.71)	
Administration Support	10/1633	ref	10/2705	ref	
Work duration^d^					
> 180 days	31/3392	1.50 (0.93, 2.40)	7/1770	0.97 (0.40, 2.33)	0.08
91–180 days	37/4866	1.10 (0.71, 1.72)	20/3191	1.39 (0.73, 2.65)	
1–90 days	40/5545	ref	17/4638	ref	
Ever had to stop work due to heat^c^					
Yes	56/5116	2.10 (1.42, 3.10)	14/2104	1.53 (0.80, 2.95)	0.56
No	48/7898	ref	23/5945	ref	
Worked near burning oil^c^					
Yes	10/1094	1.26 (0.65, 2.43)	3/978	0.78 (0.28, 2.20)	0.54
No	96/12707	ref	40/8620	ref	
Maximum overall THC exposure					
≥ 3.00 ppm	17/1819	1.99 (0.88, 4.51)	7/1504	1.27 (0.47, 3.38)	0.48
1.00–2.99 ppm	40/5018	1.42 (0.68, 2.94)	9/2165	0.92 (0.36, 2.38)	
0.30–0.99 ppm	41/4800	1.92 (0.93, 3.97)	18/2875	1.54 (0.68, 3.49)	
< 0.30 ppm	8/2151	ref	9/3041	ref	

Residential proximity to the spill is defined as living in or adjacent to a county or parish with coastline that was oiled during the spill

^a^Number of observations included in the fully adjusted model, by exposure status

^b^Risk ratios (RR) adjusting for age, gender, BMI, education, and smoking

^c^Models for the associations of stopping work due to heat with MI and working near burning oil with MI adjust for duration of clean-up work in addition to the confounders age, gender, BMI, education, and smoking

^d^For analyses of work duration stratified by residential proximity to the spill, the variable was recoded into 3 categories (1–90 days, 91–180 days, > 180 days) due to insufficient numbers of cases in the shorter work duration categories

^e^*LRT* Likelihood Ratio Test. The LRT was used to assess statistical significance of an interaction term between the modifier variable and the main exposure variable

^*^Alpha = 0.05

## Discussion

Among oil spill workers, we observed positive associations between duration of clean-up work and heat stress with self-reported MI occurring up to 1–3 years after the oil spill, and suggestive but not statistically significant associations for maximum THC exposure and decontamination work. Individuals living in proximity to the oil spill had a small, non-significantly increased risk of nonfatal MI compared to individuals living further away. The other observed associations were generally modest in magnitude and had wide confidence intervals, and conclusions should reflect the uncertainty in the observed estimates.

The positive associations between duration of clean-up work with MI, and suggestive associations between maximum THC exposure and decontamination work with MI, may be driven by chemical exposures during the oil spill response and clean-up or by other stressors related to the spill. Participants with longer work duration or tasks with more contact with fresh oil likely had higher exposures to crude oil and related exposures to volatile organic compounds, polycyclic aromatic hydrocarbons and particulate matter. Short-term increases in ambient-level particulate matter exposures increase risk of acute MI in the general population [20], and ambient exposure to volatile organic compounds is also associated with increased risk of heart disease [23, 28]. Exposures to oil-related chemicals during clean-up work may have had acute as well as longer-term impacts on incidence of MI. We did not see any association between potential work exposures to burning oil (a source of exposure to particulate matter and other combustion products) and MI, which may be due to the relatively low number of participants with exposure to burning or imprecision in the exposure measure. Workers were categorized as exposed to burning oil based on their job tasks and work locations; misclassification may have reduced our ability to detect associations with MI. It is also possible that the magnitude or duration of exposure to burning oil was not sufficient to produce an effect observable in this study.

The suggestive association between living in proximity to the oil spill and nonfatal MI may be driven by psychosocial stress caused by the spill, pollutant exposures, or other spill-related environmental factors, although the precision of the observed associations suggests that these could be chance findings. Individuals living in counties impacted by the oil spill were more likely to face economic and social hardships in the aftermath of the spill [25]. Furthermore, living in or adjacent to a county exposed to oil was associated with adverse mental health symptoms following the oil spill [26]. Psychosocial stress and anxiety contribute to hypertension [29] and other cardiovascular risk factors, and thereby may have increased risk of an acute MI or contributed to chronic development of CHD [30].

We evaluated differences in self-reported health characteristics between those living in proximity to the spill and those who live further away. Those living in proximity to the spill had a higher prevalence of self-reported fair or poor health (23.8% vs 11.6%) and higher prevalence of self-reported worse health compared to prior to the spill (36% vs 20.2%) at the time of the GuLF STUDY enrollment interview. The prevalence of self-reported hypertension prior to the oil spill was also slightly higher among those living in proximity to the spill compared to those living further away (18.7% vs 14.9%). These differences indicate poorer health among those living in proximity to the spill, and may contribute to a higher risk of MI in this group.

The present study included nonfatal MI cases occurring up to 1–3 years after the oil spill. Separate analyses for MI diagnoses reported as occurring after the active clean-up period sought to rule out the possible role of heat stress or other acute exposures on the overall results. Results restricted to the 135 events occurring on or after January 1, 2011 generally showed similar trends as our main analyses, though the association for working on clean-up and MI was stronger after the active clean-up period than for the whole study period. We were not able to further stratify the risk period due to the low number of cases. If acute stressors such as heat or physical exertion were primarily responsible for increases in nonfatal MI after the oil spill, we would have expected to see diminished associations for MI occurring after most clean-up work had ended. That some associations were actually stronger after the active clean-up period also suggests the possibility of a healthy worker effect, where events during the active clean-up period may be under-ascertained.

Stratified analyses showed possible evidence of modification by smoking and, to a lesser extent, residential proximity to the oil spill. Some associations were stronger among never smokers than among ever smokers. This may be due to reduced ability to detect spill-related associations with MI in the presence of smoking, which is a strong risk factor for MI. Stratified results also suggested some elevated risks among those living in proximity to the oil spill, though differences were generally small and not significant. Workers who lived in proximity to the oil spill likely faced greater emotional stress related to the oil spill, including loss of income and disruption of normal activities compared to those living further away, which may have contributed to a possible synergistic impact of psychosocial stress related to the oil spill and clean-up related exposures on MI.

This study has several limitations. The estimate of each individual’s maximum THC exposure is based on their task with the highest exposure and does not account for duration of that exposure or for cumulative exposure across tasks. Cumulative, chemical-specific estimates that take into account exposure from multiple tasks and duration of those tasks are currently being developed. Misclassification of workers’ THC exposure would be expected to bias results toward the null, since misclassification would likely occur non-differentially with respect to the outcome. Nonetheless, these exposure estimates were derived from detailed self-reported data on clean-up tasks and from monitoring data of airborne THC concentrations collected during the oil spill clean-up [19], providing more detailed exposure data than has been available in previous studies of oil spill workers.

Another limitation of the exposure assessment was the challenge of grouping workers based on task, due to the complex work patterns that were common among oil spill workers; many workers reported multiple tasks, even within the same time period. To examine associations with MI by work task, we categorized workers by their maximum exposure job, which may not have been the job where they spent the most time.

This study relied on self-report of MI diagnoses, which is subject to reporting errors. Research in other populations has shown that recall of an MI diagnosis may be poorer among older or less educated individuals [31], however the majority of the GuLF STUDY participants were < 60 years old at enrollment, and more than half attended at least 2 years of college. Self-report of MI has shown moderate agreement with hospital discharge data (kappa = 0.64) [32]. Furthermore, this study focused on a relatively short 1 to 3-year risk period in which participants were asked to recall the occurrence and timing of a diagnosis. We expect that misreport of MI diagnoses would be non-differential with respect to the exposures of interest and would attenuate risk estimates.

Another limitation is the lack of information on mortality in the target population during the study period. Potentially eligible individuals who died before study recruitment in 2011–2013 – due to a fatal MI or other causes – are not included in this study, as participants had to be living at the time of recruitment. Excluding individuals who did not survive to enroll in our study may have resulted in under-ascertainment of MI incidence in our study population, especially if oil spill-related exposures contributed to acute incidence of MI and fatality. We therefore may have underestimated risk of MI related to the oil spill.

The GuLF STUDY is the largest study of the human health impact of oil spills and is the first to assess heart disease among oil spill-exposed populations. The study features detailed assessment of clean-up-related tasks and improves upon exposure estimation from previous studies by developing job exposure matrix-based estimates of total hydrocarbon exposure among clean-up workers [19]. The longitudinal design of the study will allow for ongoing assessment of the observed associations over time.

## Conclusions

Results of this study suggest that several oil spill-related exposures may have been associated with an increased risk of nonfatal MI within 1–3 years after the *Deepwater Horizon* oil spill. Further research is needed to characterize the pattern of associations over time, and to better understand what specific exposures, such as stress or individual chemical exposures are driving the observed associations.
